# Dividing the Emergency Department into Red, Yellow, and Green Zones to Control COVID-19 Infection; a Letter to Editor

**Published:** 2020-05-31

**Authors:** Chee-Fah Chong

**Affiliations:** 1 School of Medicine, Fu Jen Catholic University College of Medicine, New Taipei City, Taiwan.; 2 Emergency Department, Shin-Kong Wu Ho-Su Memorial Hospital, Taipei City, Taiwan.

**Keywords:** Coronavirus Infections, Emergency Service, Hospital, Emergency Medical Services, Health Facilities, Infection Control

## Abstract

COVID-19, in certain respects, can be viewed as a CBRN (chemical, biological, radiological, or nuclear) event due to being a consequence of SARS-CoV2 virus (the “contaminant”). We, thus, reorganized our emergency department (ED) into 3 distinct zones (red, yellow, and green) for the purpose of infection control. Patients with high or medium risk of COVID-19 infection are managed in the red zones. Low-risk patients are managed in the yellow zones. All patients are prohibited to enter the green zones. Green zones are used by healthcare providers (HCPs) for personal protective equipment (PPE) donning, inventory, planning, and dining. Only HCPs who work in the red zones are required to use full level PPE (aerosol precaution). HCPs working in the yellow zones require less PPE (contact and droplet precaution). No PPE is required in the green zones. Establishing red, yellow, and green zones in the ED can be helpful in reducing cross-infections and minimizing demand for PPE.

**Dear Editor,**

Patients in our emergency department (ED) are divided into 3 groups according to their risk of COVID-19 infection. High-risk patients are those with positive TOCC (travel history, occupation, contact, cluster) who also have fever or respiratory symptoms. Persons under COVID-19 investigation (PUI) are also considered high-risk. Patients without TOCC, who have fever or respiratory symptoms, are considered medium-risk. Patients without TOCC who have no fever or respiratory symptoms are considered low-risk.

COVID-19, in certain respects, can be viewed as a CBRN (chemical, biological, radiological, or nuclear) event (1) due to being a consequence of SARS-CoV2 virus (the “contaminant”). We, thus, reorganized our ED into 3 distinct zones: red, yellow, and green zones (Figure 1) for the purpose of infection control. Patients with high or medium risk of COVID-19 infection are managed in the red zones. Red zones include the outdoor triage tents and the negative-pressure isolation room. Low-risk patients are managed in the yellow zones. Yellow zones include the indoor triage, waiting room, consultation rooms, observation rooms, and nursing station. Green zones are used by healthcare providers (HCPs) for personal protective equipment (PPE) donning, inventory, planning, and dining. All patients are prohibited from entering the green zones. 

Our spatial separation strategy using red, yellow, and green zones is also helpful in PPE conservation (2, 3). Only HCPs who work in the red zones are required to use full level PPE (aerosol precaution: N95 respirator, gown, gloves, eye protection, apron). HCPs working in the yellow zones require less PPE (contact and droplet precaution: surgical mask, gown, gloves, eye protection). No PPE is required in the green zones. Contaminated PPE should be removed before entering the green zones.

In conclusion, establishing red, yellow, and green zones in the ED can be helpful in reducing cross-infections and minimizing demand for PPE.

**Figure 1:**
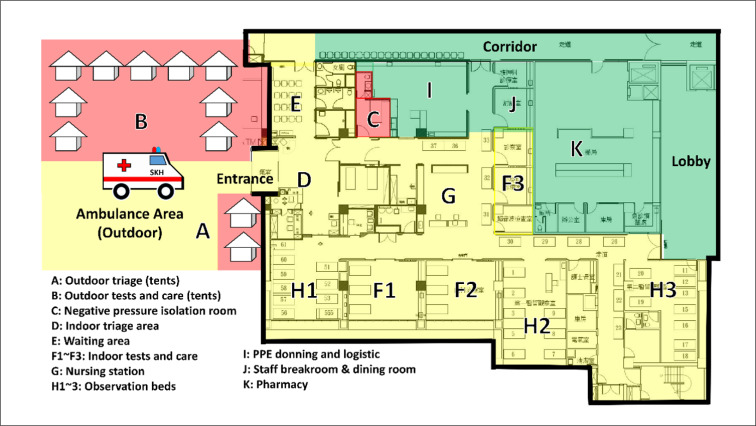
Planimetric map showing different areas of the emergency department discriminated into red, yellow, and green zones
